# Echocardiographic estimation of mean pulmonary artery pressure in critically ill patients

**DOI:** 10.1186/2036-7902-6-9

**Published:** 2014-07-02

**Authors:** Russell D Laver, Ubbo F Wiersema, Andrew D Bersten

**Affiliations:** 1 Intensive and Critical Care Unit, Flinders Medical Centre, Flinders Drive, Bedford Park, SA 5042, Australia; 2 School of Medicine, Flinders University, Sturt Road, Bedford Park, SA 5042, Australia

**Keywords:** Echocardiography, Hypertension, Pulmonary, Critical care, Intensive care

## Abstract

**Background:**

Indirect assessment of mean pulmonary arterial pressure (MPAP) may assist management of critically ill patients with pulmonary hypertension and right heart dysfunction. MPAP can be estimated as the sum of echocardiographically derived mean right ventricular to right atrial systolic pressure gradient and right atrial pressure; however, this has not been validated in critically ill patients.

**Methods:**

This prospective validation study was conducted in patients undergoing pulmonary artery catheterisation during intensive care admission. Pulmonary artery catheter (PAC) measurements of MPAP were contemporaneously compared to MPAP estimated utilising transthoracic echocardiography (TTE)-derived mean right ventricular to right atrial systolic pressure gradient added to invasively measured right atrial pressure.

**Results:**

Of 53 patients assessed, 23 had estimable MPAP using TTE. The mean difference between TTE- and PAC-derived MPAP was 1.9 mmHg (SD 5.0), with upper and lower limits of agreement of 11.6 and −7.9 mmHg, respectively. The median absolute percentage difference between TTE- and PAC-derived MPAP was 7.5%. Inter-rater reliability assessment was performed for 15 patients, giving an intra-class correlation coefficient of 0.96 (95% confidence intervals, 0.89 to 0.99).

**Conclusions:**

This echocardiographic method of estimating MPAP in critically ill patients was not equivalent to invasively measured MPAP, based on our predefined clinically acceptable range (±5 mmHg). The accuracy of this method in critically ill patients was similar to the results obtained in ambulatory patients and compared favourably with regard to the accuracy with echocardiographic estimation of systolic pulmonary arterial pressure. The utility of this technique is limited by frequent inability to obtain an adequate tricuspid regurgitant time-velocity integral in critically ill patients.

## Background

Pulmonary hypertension (PH) is a common problem in critically ill patients and is associated with right heart dysfunction and increased morbidity and mortality [1]. Historically, the intensive care physician has used the pulmonary artery catheter (PAC) to measure pulmonary pressures. Although still considered the reference standard for diagnosis and quantification of PH in the critically ill [2,3], growing concerns regarding the risk of complications and lack of demonstrated outcome benefit with PAC-guided haemodynamic monitoring have led to a reduction in PAC use over recent years [4].

The increased availability of point-of-care ultrasound machines in the intensive care unit (ICU) has facilitated the use of echocardiography as an alternative to the PAC for the evaluation of the haemodynamically unstable patient. Estimation of systolic pulmonary arterial pressure (SPAP) from the peak tricuspid regurgitant velocity is the most widely used echocardiographic measure of PH severity and has been validated in a broad range of clinical situations, although concerns about accuracy remain [5]. Mean pulmonary arterial pressure (MPAP), however, is the preferred measure to diagnose, assess severity and determine response to therapy in PH [6]. Previous echocardiographic methods of estimating MPAP require the presence of pulmonary regurgitation and precise time interval measurements or are based on empiric formulas extrapolated from SPAP estimates, making them poorly suited to the ICU environment [5,7-10].

Recently, Aduen and colleagues described a new, simpler echocardiographic method of estimating MPAP calculated as mean right ventricular to right atrial (RV-RA) systolic pressure gradient plus right atrial pressure (RAP) [6]. Mean RV-RA gradient was estimated from the tricuspid regurgitant time-velocity integral, obtained from the continuous wave Doppler interrogation of the tricuspid regurgitant jet. RAP was estimated echocardiographically. The study demonstrated satisfactory correlation between the echocardiographic estimate and PAC data but was confined to ambulatory patients. Critically ill patients may differ due to acute changes in pulmonary pressures, effects of positive intra-thoracic pressure (mechanical ventilation) and difficulties in obtaining acoustic windows.

Our aim in the present study was to establish the accuracy of this new echocardiographic method of MPAP estimation in a critically ill patient population. Furthermore, we attempted to increase the accuracy of our results by direct measurement of RAP, rather than echocardiographic estimation with its associated inaccuracies [5,11].

## Methods

### Setting

This prospective validation study was conducted at Flinders Medical Centre Intensive and Critical Care Unit, South Australia, a large metropolitan tertiary ICU, between February 2011 and February 2012. The study was approved by the Flinders Clinical Research Ethics Committee, Southern Adelaide Health Service (450.10). Informed consent was gained from all patients or next of kin where appropriate.

### Patients

Consecutive patients aged 18 years and over admitted to the ICU who had a PAC in situ were assessed for eligibility. Exclusion criteria were patient refusal, inability to obtain informed consent or patient factors making transthoracic echocardiography (TTE) assessment impossible. Patient demographics were recorded.

### Echocardiographic examination

A Sonosite M-Turbo® ultrasound machine with phased array (1 to 5 MHz) transducer was used for echocardiographic assessments. Patients were assessed in the supine or semi-recumbent position. Limited TTE was performed to estimate MPAP and SPAP from the tricuspid regurgitant jet. The view providing the most complete envelope, with the highest-velocity regurgitant jet, was used for measurements.

**Figure 1:**
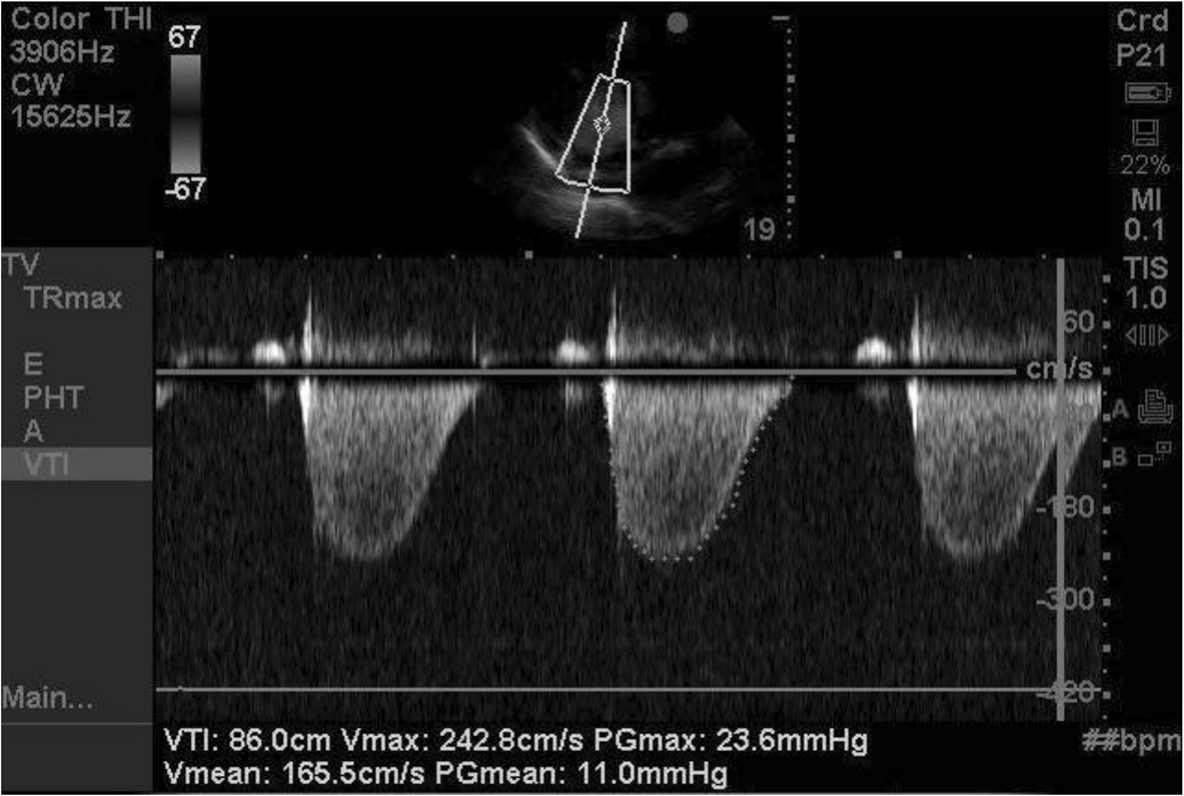
**Echocardiographic estimation of pulmonary arterial pressures.** Estimation of peak and mean right ventricular to right atrial systolic pressure gradients utilising continuous wave Doppler of the tricuspid regurgitant jet. Peak and mean pressure gradients were added to invasively measured right atrial pressures to estimate systolic and mean pulmonary artery pressures, respectively.

The average of three each of peak and mean RV-RA systolic gradients was calculated and recorded. Peak RV-RA gradient was estimated using the modified Bernoulli equation [5]. Mean RV-RA systolic gradient was estimated using the method described by Aduen and colleagues [6]. SPAP and MPAP were calculated by adding the PAC-derived RAP to peak and mean RV-RA gradients, respectively. All measurements were obtained at end expiration (Figure 1).

All assessments were performed by the same echocardiographer (RL, an advanced trainee in intensive care medicine). When available, a second echocardiographer (UW, a consultant intensivist) performed an assessment immediately afterwards, allowing an estimation of inter-rater reliability. The primary echocardiographer was blinded to PAC measurements. The second echocardiographer was blinded to the primary echocardiographer's TTE measurements.

### Pulmonary artery catheter

The decision to utilise a PAC to guide patient management was determined independently by the treating intensivist. PAC data was recorded during or immediately after echocardiographic assessment. The transducer was zeroed and levelled to the level of the right atrium, taken as 5 cm vertically below the manubriosternal junction, thus minimising the effect of differences in patient position or anteroposterior (AP) chest wall diameter [12].

Measurements obtained from the PAC included SPAP, diastolic pulmonary arterial pressure (DPAP), RAP and pulmonary artery occlusion pressure (PAOP). MPAP was calculated as (SPAP − DPAP) / 3 + DPAP [13]. An average of three measurements was taken at end expiration, with the patient supine or semi-recumbent (unchanged from TTE assessment).

Cardiac index was calculated as cardiac output (most recent measurement, assessed by thermodilution) divided by body surface area [14]. Other measurements recorded included heart rate, rhythm and systemic mean arterial pressure (MAP).

### Statistical analysis

Utilising the concept of equivalence [15], it was estimated that an adequately powered trial would consist of 25 patients with 95% confidence intervals for a clinically acceptable difference of ±5 mmHg between the gold standard (PAC) and TTE-determined MPAP.

The mean and standard deviation (SD) of the differences between PAC- and TTE-derived measurements of MPAP is reported. Upper and lower limits of agreement (i.e. 2SD) were calculated using Bland-Altman analysis [16], assuming normal distribution of differences between PAC- and TTE-derived measurements. A priori subgroup analysis was performed for mechanically ventilated and spontaneously ventilating patients. Absolute percentage differences were calculated as the absolute value of the difference between TTE- and PAC-derived measurements divided by the PAC-derived measurement and expressed as a percentage.

Data are expressed as number (percentage) for categorical variables and mean (±SD) for continuous variables where appropriate. Independent samples *t* tests were used to determine the relationship between continuous and categorical variables. Chi-squared test for independence was used to determine the relationship between categorical variables (Fisher's exact test used when values in any cell are <5). Inter-rater reliability was assessed using intra-class correlation analysis, utilising a two-way mixed model (single measures) with absolute agreement for measurements. SPSS version 19.0 was used for all statistical analysis.

## Results

**Figure 2:**
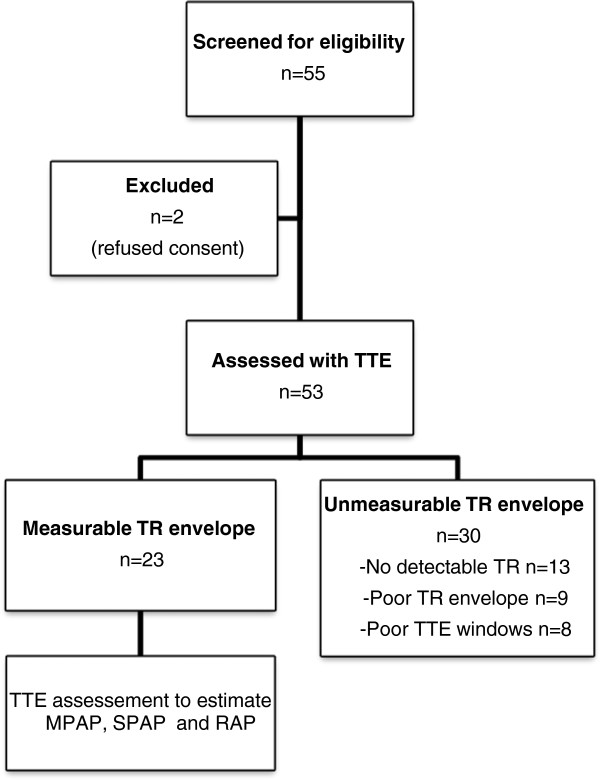
Patient flow diagram.

A total of 55 patients were screened for study eligibility. Two patients declined participation, leaving 53 patients who were assessed echocardiographically. A further 30 patients were excluded due to inability to obtain a measurable tricuspid regurgitation (TR) envelope. Of those with unmeasurable TR, 13 patients had no detectable TR (43%), 9 had a poor-quality regurgitant envelope (30%) and 8 had poor acoustic windows (27%). The final 23 patients underwent TTE examination to determine MPAP, SPAP and RAP echocardiographically (Figure 2).

Patient demographics for the 53 patients who underwent TTE assessment are shown in Table 1. Characteristics significantly associated with unmeasurable TR envelope were increased chest wall diameter, male gender and mechanical ventilation. Haemodynamic variables measured from the 53 patients who underwent TTE assessment are shown in Table 2. Haemodynamic variables significantly associated with unmeasurable TR envelope were lower SPAP and PAOP and higher MAP.

**Table 1:** Patient demographics

	**Measurable TR envelope (*n*** **= 23)**	**Unmeasurable TR envelope (*n*** **= 30)**	***p *value**^**a**^	**All participants (*n*** **= 53)**
Age (years), mean (SD)	65.4 (16.7)	66.3 (15.1)	0.823	65.9 (15.7)
Male, *n* (%)	9 (39%)	22 (73%)	0.026	31 (59%)
BMI, mean (SD)	26.4 (4.3)	28.4 (6.0)	0.178	27.5 (5.4)
Chest wall AP diameter (cm), mean (SD)	20 (2.6)	22.3 (3.3)	0.008	21.32 (3.25)
Diagnostic category, *n* (%)				
Cardiothoracic surgery	9 (39%)	11 (37%)		20 (38%)
Cardiac	8 (35%)	11 (37%)		19 (36%)
Sepsis	3 (13%)	5 (17%)		8 (15%)
Liver transplant	1 (4%)	2 (7%)		3 (6%)
Other	2 (9%)	1 (3%)	0.920	3 (6%)
APACHE IIIj, mean (SD)	81.0 (24.4)	79.5 (25.9)	0.840	80.2 (25)
ICU length of stay at time of assessment (days), mean (SD)	3.9 (3.7)	4.6 (4.3)	0.537	4 (4)
PaO_2_:FiO_2_, mean (SD)	264.9 (117.5)	236.2 (85.7)	0.454	244.5 (94.8)
Mechanically ventilated, *n* (%)	9 (39%)	22 (73%)	0.026	31 (59%)
Mode of mechanical ventilation, *n* (%)				
SIMV + PSV	1 (11%)	5 (23%)		6 (19%)
PSV	8 (89%)	17 (77%)	0.642	25 (81%)

TR, tricuspid regurgitation; BMI, body mass index; AP, anteroposterior; APACHE IIIj, acute physiology and chronic health evaluation version IIIj; ICU, intensive care unit; PaO_2_, partial pressure of arterial oxygen; FiO_2_, fraction of inspired oxygen; SIMV, synchronised intermittent mandatory ventilation; PSV, pressure support ventilation. ^a^Comparing measurable vs unmeasurable TR envelope.

**Table 2:** Haemodynamic measurements

	**Measurable TR envelope (*n*** **= 23)**	**Unmeasurable TR envelope (*n*** **= 30)**	***p *value**^**a**^	**All participants (*n*** **= 53)**
SPAP (mmHg)	49.6 (15.5)	41.7 (10.7)	0.034	45.1 (13.5)
MPAP (mmHg)	30.2 (9.3)	28.7 (6.5)	0.525	29.4 (7.8)
RAP (mmHg)	11.8 (6.1)	11.7 (3.2)	0.987	11.7 (4.6)
PAOP (mmHg)	19.8 (4.4)	16.6.(5.2)	0.021	18.0 (5.1)
CI (L/min/m^2^)	2.9 (1.0)	2.9 (0.9)	0.823	2.9 (0.9)
Heart rate (bpm)	89 (20)	89 (18)	0.873	89 (18)
MAP (mmHg)	78 (9)	85 (14)	0.043	82 (12)

Data are expressed as mean (SD). TR, tricuspid regurgitation; PAC, pulmonary arterial catheter; SPAP, systolic pulmonary arterial pressure; MPAP, mean pulmonary arterial pressure; RAP, right atrial pressure; PAOP, pulmonary artery occlusion pressure; CI, cardiac index; MAP, mean arterial pressure. ^a^Comparing measurable vs unmeasurable TR envelope.

**Figure 3:**
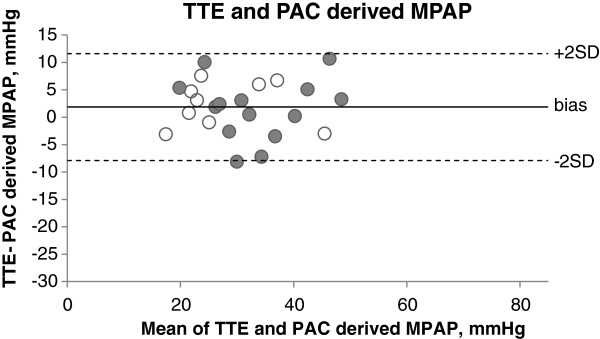
**Bland-Altman plot of TTE- and PAC-derived MPAP.** The bias between TTE- and PAC-derived MPAP was 1.9 mmHg (SD 5.0), with upper and lower limits of agreement of 11.6 and −7.9 mmHg. White marker: mechanically ventilated, shaded marker: spontaneously ventilating.

**Figure 4:**
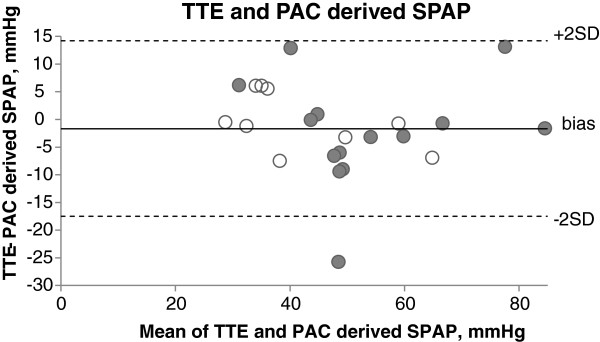
**Bland-Altman plot of TTE- and PAC-derived SPAP.** The bias between TTE- and PAC-derived SPAP was −1.7 mmHg (SD 8.1), with upper and lower limits of agreement of 14.2 and −17.5 mmHg, respectively. White marker: mechanically ventilated, shaded marker: spontaneously ventilating.

In patients with a measurable TR envelope, the apical 4-chamber was the most frequently obtained acoustic window to demonstrate TR and was most commonly used for measurements. The subcostal 4-chamber was the least frequently obtained and least commonly used for measurements (Table 3).For the primary echocardiographer, the bias between TTE- and PAC-derived MPAP was 1.9 mmHg (SD 5.0), with upper and lower limits of agreement of 11.6 and −7.9 mmHg, respectively (Figure 3). The median absolute percentage difference between TTE- and PAC-derived MPAP was 7.5%. For mechanically ventilated patients, the bias between TTE- and PAC-derived MPAP was 2.4 mmHg (SD 4.1), with upper and lower limits of agreement of 10.4 and −5.6 mmHg, respectively. For spontaneously ventilating patients, the bias between TTE- and PAC-derived MPAP was 1.5 mmHg (SD 5.6), with upper and lower limits of agreement of 12.4 and −9.4 mmHg, respectively.The bias between TTE- and PAC-derived SPAP was −1.7 mmHg (SD 8.1), with upper and lower limits of agreement of 14.2 and −17.5 mmHg, respectively (Figure 4). The median absolute percentage difference between TTE- and PAC-derived SPAP was 1.9%.

**Table 3:** Acoustic windows

**View**	**Tricuspid regurgitation demonstrated (%)**	**Used for measurement (%)**
Parasternal long axis-right chamber view	85	12.8
Parasternal short axis-level of aortic valve	82	12.8
Apical 4-chamber	97	71.8
Subcostal 4-chamber	38	2.6

For the 15 patients examined by both echocardiographers, inter-rater reliability assessment was performed, giving an intra-class correlation coefficient of 0.96 (95% confidence intervals, 0.89 to 0.99).

## Discussion

Echocardiographic estimation of MPAP was not equivalent to invasively measured MPAP according to our narrow predetermined limits of ±5 mmHg. The bias between TTE- and PAC-derived MPAP was small at 1.9 mmHg; however, upper and lower limits of agreement (11.6 and −7.9 mmHg) lay outside our clinically acceptable range. The limits of agreement between TTE- and PAC-derived MPAP were similar for mechanically ventilated and spontaneously ventilating patients, but the study was underpowered to detect a significant difference between these two groups.

Our results were similar to those reported by Aduen and colleagues (bias between TTE-estimated and PAC-measured MPAP of −1.6 mmHg, with upper and lower limits of agreement of −16.6 to 13.7 mmHg, respectively) [6]. The improved accuracy of MPAP estimation in our study, as suggested by narrower limits of agreement, may be attributed to the use of invasively measured RAP.

Inter-rater reliability for the 15 patients who were examined by both echocardiographers was excellent (intra-class correlation coefficient of 0.96); however, only the primary echocardiographer was blinded to PAC measurements, limiting interpretation of this result.

Major strengths of this study included contemporaneous TTE and PAC assessments in a heterogenous group of critically unwell patients and direct measurement of RAP. Invasively measured RAP minimised the effect of any error in phlebostatic axis determination on MPAP measurement. The original trial by Aduen and coworkers used mid-thorax level as the phlebostatic axis, which may have affected the measurements obtained.

The PAC was used as the reference standard. Dynamic response and reproducibility of the pulmonary arterial waveform are known to vary considerably with PAC [17]. Ideally dynamic calibration would have been undertaken prior to measurements, but this would have increased the complexity of the study substantially and mean pressure is a relatively robust measure, less affected by dynamic response.

Heart rate was not shown to affect accuracy of MPAP estimation. In patients with atrial fibrillation (AF), it was noted that there was large beat-to-beat variability in the magnitude of the tricuspid regurgitant jet and pulmonary arterial pressure trace. Eight of 23 patients (35%) were in AF at the time of echocardiographic assessment. When subjects were analysed according to their underlying heart rhythm, the groups were not dissimilar, although the study was underpowered to detect significant differences. Determining the accuracy of this method of MPAP estimation in patients with AF would require averaging measurements over a larger number of cardiac cycles or ideally simultaneous TTE and PAC analysis for a single cardiac cycle.

A significant limitation of the study was the inability to estimate MPAP echocardiographically in the majority of patients screened. Only 23 of 53 patients (43%) assessed echocardiographically had adequately visualised TR to allow estimation of MPAP, leaving the study underpowered. A previous study by Bouhemad and colleagues observed the presence of TR in 85% of hypoxemic mechanically ventilated critically ill patients [10]. It is likely that satisfactory imaging could have been obtained in more patients had they been optimally positioned and a higher-end echocardiography machine used. However, the point-of-care device used was representative of the technology available in the ICU for repeated TTE assessments by clinicians at the bedside. Agitated saline may have improved the quality of the TR envelope to allow estimation of MPAP in selected cases. Agitated saline was not used in our study as it was not demonstrated to improve MPAP estimation in the original trial [6]; however, it has been shown to improve accuracy of SPAP measurement [18]. Transesophageal echocardiography may also have a role, given the difficulties in obtaining acoustic windows in critically ill patients utilising TTE.

Increased AP chest wall diameter, mechanical ventilation and male gender were associated with reduced ability to obtain MPAP estimates. Difficulty obtaining MPAP estimates in men may be secondary to increased AP chest wall diameter (mean male AP chest wall diameter was 23.3 vs 18.5 cm for females). It was expected that TTE assessment would be more accessible in medical patients, as opposed to post-cardiothoracic surgical patients [19-21]. Post-cardiothoracic surgery patients were no less likely to have measurable TR than other diagnostic groups. However, only patients in the cardiothoracic surgical group had unmeasurable TR due to poor acoustic windows (8 of 20 cardiothoracic surgical patients assessed, 40%).

Haemodynamic variables associated with inability to estimate MPAP included lower SPAP and PAOP and higher MAP. Lower SPAP was predictably associated with reduced ability to measure TR, as increased SPAP is known to be associated with increased severity of TR [22]. There was a non-significant trend towards reduced ability to estimate MPAP at lower measured MPAP. Higher PAOP may also increase TR secondary to elevations in pulmonary arterial pressure and right ventricular afterload.

This method of echocardiographic estimation of MPAP has not, at this time, entered into routine clinical practice. Subsequent study has demonstrated similar accuracy and precision to formulas deriving MPAP from SPAP [23]. As stated in the original trial by Aduen and coworkers, this alternative estimate of MPAP was comparable in reliability and accuracy with traditional SPAP estimation. TTE-estimated SPAP compared to estimated MPAP had a similar bias (−1.7 vs 1.9 mmHg) and smaller median absolute percentage difference between invasive measures (1.9% vs 7.5%), but as expected (due to higher absolute numbers), limits of agreement were wider for SPAP estimation. Given the comparable accuracy of TTE-estimated SPAP and MPAP, there may be a role for echocardiographic estimation of MPAP using this technique as part of a comprehensive right heart assessment, rather than as a stand-alone measure.

## Conclusions

Transthoracic echocardiographically estimated mean pulmonary artery pressure utilising mean right ventricular to right atrial systolic pressure gradient added to invasively measured right atrial pressure in critically ill patients was not equivalent to invasively measured mean pulmonary arterial pressure from right heart catheterisation based on a predefined clinically acceptable range of ±5 mmHg. The accuracy of this method in critically ill patients was comparable to results obtained in ambulatory patients [6] and compared favourably with regard to accuracy with echocardiographic estimation of systolic pulmonary arterial pressure. Requirement for the presence of tricuspid regurgitation and an acceptable Doppler signal may limit application of this technique.

## Abbreviations

AP: anteroposterior; APACHE IIIj: acute physiology and chronic health evaluation version IIIj; BMI: body mass index; CI: cardiac index; DPAP: diastolic pulmonary arterial pressure; ICU: intensive care unit; MAP: mean arterial pressure; MPAP: mean pulmonary arterial pressure; PAC: pulmonary artery catheter; PAOP: pulmonary artery occlusion pressure; PH: pulmonary hypertension; PSV: pressure support ventilation; RAP: right atrial pressure; RV-RA: right ventricular to right atrial; SD: standard deviation; SIMV: synchronised intermittent mandatory ventilation; SPAP: systolic pulmonary arterial pressure; TR: tricuspid regurgitation; TTE: transthoracic echocardiography.

## Competing interests

The authors declare that they have no competing interests.

## Authors’ contributions

RL contributed to study design, data acquisition, analysis and interpretation, and drafting and revising of the submitted manuscript. UW conceived the study, contributed to study design, data acquisition and interpretation, and drafting and revising of the submitted manuscript. AB contributed to study design, data interpretation and drafting and revising of the submitted manuscript. All authors have read and approved the final manuscript. RL takes responsibility for (is the guarantor of) the content of the manuscript, including the data and analysis.
